# Association between health literacy and dysphagia in the community-dwelling older population: a cross-sectional study

**DOI:** 10.1007/s40520-023-02499-4

**Published:** 2023-07-28

**Authors:** Huafang Zhang, Chenxi Ye, Simei Zhang, Dan Yang, Xiaolan Gong, Sihan Li, Wenfeng Xue, Jie Su, Lancai Zhao, Yufeng Qiu, Xiaona He, Yongming Zhang, Mengling Tang

**Affiliations:** 1 The Fourth Affiliated Hospital, Zhejiang University School of Medicine, Yiwu, 322000 China; 2 Department of Public Health, Fourth Affiliated Hospital, Zhejiang University School of Medicine, Hangzhou, 310058 China; 3 Zhejiang Nursing Association, Hangzhou, 310003 China; 4 Futian Community Health Service Center, Yiwu, 322000 China

**Keywords:** Dysphagia, Health literacy, Older adult, EAT-10, Water swallow test

## Abstract

**Background:**

Dysphagia, or swallowing disorders, has become a growing concern due to the aging population, and health literacy plays a crucial role in active aging. However, the relationship between them remains unclear.

**Aims:**

To investigate the association between health literacy and dysphagia among community-dwelling older adults in China.

**Methods:**

A survey was conducted on 4462 older adults aged 65 and above in a community in Yiwu City, China, from May 2021 to January 2022. Swallowing problems were assessed using a 30 ml water swallowing test (WST) and the Eating Assessment Tool-10 questionnaire (EAT-10). The participants' health literacy was evaluated using the Chinese Health Literacy Scale (CHLS). Logistic regression and t tests were employed to measure the association between them.

**Results:**

The prevalence of dysphagia was 5.70% and 7.85% as determined by EAT-10 and 30 ml-WST, respectively. The health literacy level of community-dwelling older adults was 24.4 ± 4.93 (9–45). Participants with dysphagia exhibited lower levels of health literacy (*p* < 0.05). The logistic regression model demonstrated an inverse association between health literacy and dysphagia (OR = 0.94, 95%CI = 0.91–0.96 for EAT-10, and OR = 0.93, 95%CI = 0.92–0.95 for WST). Moreover, this association remained significant even after adjusting for covariates.

**Discussion:**

Older adults with dysphagia have lower levels of health literacy, particularly in terms of their ability to seek medical advice, acquire and evaluate medical information, and access social support resources.

**Conclusions:**

Health literacy is associated with dysphagia among community-dwelling older adults. Effective interventions should be implemented to provide support in terms of both medical services and social support for this population.

**Supplementary Information:**

The online version contains supplementary material available at 10.1007/s40520-023-02499-4.

## Introduction

Aging poses a significant global challenge, and China is currently witnessing a rise in the number and proportion of older adults within its population [1]. By the end of 2021, the population of individuals aged 65 and above in China exceeded 200 million, accounting for 14.2% of the total population [1]. This demographic shift has emerged as a pressing public health issue. Despite advancements in life expectancy, the duration of healthy living has not shown a commensurate increase [2]. Dysphagia, which refers to impairment of the swallowing process [3], has emerged as a growing health issue among the aging population. Current evidence suggests that dysphagia affects 11.4% to 25.1% of community-dwelling older adults, with a prevalence of up to 53.8% among those who cannot live independently [4–8]. Moreover, the prevalence of dysphagia steadily increases with age and the presence of comorbidities [9]. Dysphagia is associated with various detrimental health consequences, including a higher risk of pneumonia and malnutrition [10], diminished well-being, and reduced likelihood of survival [11]. These implications impose a significant burden on families, the healthcare system, and society as a whole. Therefore, early identification and intervention for dysphagia are crucial to support older adults in achieving active aging.

An increasing body of research has focused on the role of health literacy in the context of aging. Health literacy (HL) was initially proposed in the 1970s and defined by the World Health Organization (WHO) as “the cognitive and social skills that determine individuals' motivation and ability to access, understand, and use information in ways that promote and maintain good health” [12]. Over the years, the concept of HL has expanded from personal abilities in reading, writing, and comprehending health information to a social practice involving interactions with the healthcare system [12]. Given the rise in the older population and average life expectancy, healthy aging has become a key focus for the global community. Evidence indicates that higher levels of HL enable older adults to maintain better health and well-being [13]. Conversely, low HL is associated with increased hospital admissions, prolonged hospital stays [14], and a higher likelihood of mortality [15].

As a geriatric syndrome, dysphagia requires early identification and intervention to manage its associated complications, such as malnutrition, pneumonia, and dehydration, and to improve the decreased quality of life resulting from the condition. Apart from pathological factors, the occurrence and worsening of dysphagia are influenced by subjective factors, including older adults' awareness and knowledge of the condition, adherence to dysphagia management recommendations, and more. Research has indicated that many older adults are unaware of their swallowing difficulties [16], and even among those who self-report dysphagia, nearly half of them do not seek medical advice or services [17]. Furthermore, evidence suggests low adherence to dysphagia-specific recommendations [18]. These factors pose barriers to the management of dysphagia in the community. Therefore, this study aimed to explore the relationship between HL and dysphagia among the aging population residing in the community. The findings aim to provide additional insights for the improved management of dysphagia in older adults within the community setting.

## Methods

### Study design and participants

Participants were recruited from the community health service center, where they had come for health examinations provided by the local government. Individuals aged 65 and above were invited to participate in a face-to-face interview using a structured questionnaire. Those who were able to cooperate and complete the investigation were included, while older adults with hearing impairment, inability to understand Mandarin, or refusal to participate were excluded. Investigators were well-trained nurses from the Fourth Affiliated Hospital, Zhejiang University School of Medicine, and medical students from Zhejiang University School of Medicine. Data collection took place between May 2021 to January 2022 at the Futian Community Health Service Center in Yiwu City. A total of 4,701 older adults aged 65 and above were approached, and 239 individuals were excluded due to hearing impairment, inability to understand Mandarin, or refusal to participate. Consequently, a total of 4,462 older adults were recruited.

### Dysphagia screening

To screen for swallowing problems, the Eating Assessment Tool-10 (EAT-10) and the 30 ml Water Swallowing Test (WST) were employed. The EAT-10 [19] is a subjective perception questionnaire comprising 10 items that assess various aspects of the swallowing process, emotional experiences during swallowing, and the impact of swallowing problems on daily life. Each item is rated on a scale from 0 to 4, with 0 indicating no problem and 4 indicating a severe problem. In our study, dysphagia was defined as a total score of 3 or higher on the EAT-10 questionnaire. Older adults self-evaluated the EAT-10 questionnaire, and their responses were recorded by the investigators.

The WST involved participants drinking 30 ml of water consecutively while in a seated position. Observations were made regarding clinical behaviors, such as coughing and choking, as well as the quantity of water swallowed and the time taken. The outcomes were graded as follows: Grade 1 indicated drinking the water in one attempt (or within 5 s) without choking or coughing; Grade 2 denoted drinking the 30 ml of water more than 2 times (or more than 5 s) without choking or coughing; Grade 3 indicated choking or coughing when drinking once or within 5 s; Grade 4 referred to drinking more than 2 times (or more than 5 s) with choking or coughing; Grade 5 indicated frequent coughing and the inability to finish the drink. Participants with values ranging from 2 to 5 were classified as having dysphagia. The WST is a widely used objective assessment tool for the preliminary screening of swallowing problems and determining their risk level [20].

### Health literacy measurement

To measure the HL of community-dwelling older adults, the Chinese Health Literacy Scale (CHLS) was employed. We developed and validated the CHLS based on the Health Literacy Questionnaire (HLQ) by Osborne et al. in [21]. The CHLS demonstrated a Cronbach's α coefficient of 0.86 [22]. Comprising 9 items, each with 5 response options (strongly disagree, disagree, unclear/refuse to answer, agree, strongly agree), the CHLS assessed participants' agreement with various statements. Responses were graded on a scale of 1 (strongly disagree) to 5 (strongly agree). The total score ranged from 9 to 45, with higher scores indicating higher levels of HL. Through exploratory factor analysis (EFA), the 9 items were divided into 3 dimensions: D1—Ability to Seek Medical Advice (4 items, total score ranging from 4 to 20); D2—Seeking and Appraising Medical Information (2 items, total score ranging from 2 to 10); and D3—Social Resource Support (3 items, total score ranging from 3 to 15) (details are provided in supplementary materials Table S1).

### Covariates

Demographic variables including gender, age, body mass index (BMI), marital status, and educational level were collected. Additional information on smoking status, alcohol drinking status, the number of chronic diseases, and history of pneumonia or falls within the previous year was also recorded. BMI measurements were obtained by healthcare professionals at the health service center using a calibrated electronic weight meter with height measurement function during the interview. Other covariates were self-reported by older adults and their responses were recorded by the investigators. BMI categories were classified as underweight (BMI < 18.5), normal weight (BMI = 18.5–23.9), overweight (BMI = 24.0–27.9), and obesity (BMI ≥ 28.0). Educational level was categorized into options of no formal schooling, primary school, middle school, high school, and college or above. Smoking and alcohol drinking status were categorized as smokers/non-smokers and alcohol consumers/non-consumers, respectively. Smokers were defined as individuals who smoked 1 or more cigarettes per day for more than 3 months, while alcohol consumption was defined as consumption of 20 ml of alcohol per week for more than 3 months. Participants were asked to report their history of chronic diseases, and the number of self-reported chronic diseases was calculated. Cardiovascular diseases included hypertension, coronary heart disease, and hypotension. Endocrine system diseases included diabetes mellitus, thyroid disease, and osteoporosis. Nervous system diseases included Parkinson’s disease, epilepsy, aphasia, Alzheimer's disease, and stroke. Respiratory diseases included asthma, chronic obstructive pulmonary disease, chronic bronchitis, and pulmonary tuberculosis. If participants reported other diseases not listed above, they were noted as having other diseases, and the specific name was recorded. The presence of chronic diseases was categorized into three levels: none, single, or multiple chronic diseases. Participants were asked to recall any history of pneumonia or falls within the previous year.

### Statistical analysis

The study population was not excluded based on missing data in the general population description. However, data with missing values were excluded from subsequent data analysis. Participant characteristics were presented as mean ± standard deviation (SD) for continuous variables and frequency (percentage) for categorical variables. Univariate analyses comparing HL and dysphagia were performed using t-tests. Logistic regression models were used to estimate the odds ratios (ORs) of dysphagia along with their corresponding 95% confidence intervals (CIs). Multivariable models were adjusted for the following variables: Model I, adjusted for age and gender; Model II, further adjusted for marital status and educational level; Model III, additionally adjusted for smoking status, alcohol drinking status, BMI, number of chronic diseases, and history of pneumonia or falls within the previous year. Subgroup analyses were conducted by gender, age, BMI, marital status, educational level, smoking status, alcohol drinking status, and number of chronic diseases, adjusting for potential confounding risk factors including age, gender, marital status, educational level, smoking status, alcohol drinking status, BMI, number of chronic diseases, and history of pneumonia or falls within the previous year. Statistical significance was considered if p ≤ 0.05. Data were analyzed using R software version 4.1.1.

## Results

### Demographic and health-related characteristics

A total of 4,462 older adults were included in this study, with a mean age of 73.22 ± 6.23, 48.97% were men and 51.03% were women. Among the participants, 77.35% were married, and 70.67% had a primary school education or higher. 83.63% of the participants had more than one chronic disease. The prevalence of smoking and alcohol consumption among the participants was 29.77% and 36.22%, respectively, and 86.15% denied a history of falls in the past year. Among the 4,462 participants, 59 participants with incomplete information in EAT-10 and 90 with incomplete information in WST were excluded, resulting in a dysphagia prevalence of 5.70% (251/4,403) and 7.85% (343/4,372) based on EAT-10 and WST screening, respectively. After excluding 61 participants with incomplete information in CHLS, 4401 participants answered the CHLS, with an average score of 24.4 ± 4.93. The characteristics of the participants are summarized in Table 1.

**Table 1:** Characteristics of the participants

Characteristics	N (%)/mean ± SD
Gender	
Male	2185 (48.97%)
Female	2277 (51.03%)
Marital status	
Married	3446 (77.35%)
Widowed and others	1009 (22.65%)
Educational level	
No formal schooling	1306 (29.33%)
Primary school	1876 (42.13%)
Middle school or above	1271 (28.54%)
Smoking status	
Yes	1324 (29.77%)
No	3124 (70.23%)
Alcohol drinking status	
Yes	1605 (36.22%)
No	2826 (63.78%)
History of pneumonia within the previous year	
Yes	95 (2.16%)
No	4313 (97.84%)
History of falls within the previous year	
Yes	610 (13.85%)
No	3795 (86.15%)
Number of chronic diseases	
None	729 (16.37%)
Single	1457 (32.72%)
Multiple	2267 (50.91%)
EAT-10	
Without dysphagia (≤ 2)	4152 (94.30%)
With dysphagia (≥ 3)	251 (5.70%)
WST	
Without dysphagia (Grade1)	4029 (92.15%)
With dysphagia (Grade2 ~ 5)	343 (7.85%)
Age (years)	73.22 ± 6.23
BMI (kg/m^2^)	23.4 ± 3.27
Health literacy	
Total	24.4 ± 4.93
D1*	11.7 ± 2.28
D2*	5.08 ± 1.47
D3*	7.67 ± 1.96

*D1: Ability to seek medical advice

D2: Seeking and appraising medical information

D3: Social resource support

### Association between health literacy and dysphagia

Univariate analysis revealed a significant relationship between dysphagia (screened by EAT-10 and WST) and HL. Participants with dysphagia had lower HL scores (22.70 ± 4.96 vs. 24.50 ± 4.88, p < 0.05 for EAT-10; 22.08 ± 5.90 vs. 24.60 ± 4.78, p < 0.05 for WST). The results are presented in Table 2.

**Table 2:** Univariate analysis of health literacy and dysphagia

Characteristics	EAT-10				WST		
	Without dysphagia mean ± SD	With dysphagia mean ± SD	p value	Without dysphagia mean ± SD		With dysphagia mean ± SD	p value
Health literacy (total)	24.50 ± 4.88	22.70 ± 4.96	< 0.05	24.60 ± 4.78		22.80 ± 5.90	< 0.05
D1: Ability to seek medical advice	11.70 ± 2.27	11.00 ± 2.29	< 0.05	11.80 ± 2.22		10.90 ± 2.67	< 0.05
D2: Seeking and appraising medical information	5.11 ± 1.46	4.59 ± 1.46	< 0.05	5.13 ± 1.43		4.59 ± 1.78	< 0.05
D3: Social resource support	7.71 ± 1.95	7.14 ± 1.97	< 0.05	7.71 ± 1.92		7.27 ± 2.29	< 0.05

Logistic regression analysis further demonstrated an inverse association between HL and dysphagia. The OR and 95% CIs for HL and the risk of dysphagia are presented in Table 3. Both the univariate model and adjusted model indicated that participants with higher HL were less likely to have dysphagia, as screened by EAT-10 (adjusted OR = 0.95; 95% CIs 0.93–0.97) and WST (adjusted OR = 0.96; 95% CIs 0.94–0.98). The significance persisted after controlling for demographic and health covariates, except in the “Social Resource Support” dimension when using WST as the screening tool.

**Table 3:** Association between health literacy and dysphagia

Health literacy	OR(95%CI)							
	EAT-10				WST			
	Crude^a^	Model I^b^	Model II^c^	Model III^d^	Crude^a^	Model I^b^	Model II^c^	Model III^d^
Total	0.94 (0.91, 0.96)	0.95 (0.92, 0.97)	0.95 (0.92, 0.97)	0.95 (0.93, 0.97)	0.93 (0.92, 0.95)	0.96 (0.94, 0.98)	0.96 (0.94, 0.98)	0.96 (0.94, 0.98)
D1*	0.88 (0.84, 0.93)	0.90 (0.86, 0.95)	0.91 (0.86, 0.96)	0.90 (0.86, 0.96)	0.87 (0.83, 0.91)	0.91 (0.87, 0.95)	0.91 (0.87, 0.96)	0.92 (0.87, 0.96)
D2*	0.80 (0.73, 0.86)	0.82 (0.76, 0.90)	0.83 (0.76, 0.90)	0.85 (0.78, 0.93)	0.79 (0.73, 0.85)	0.84 (0.78, 0.91)	0.85 (0.79, 0.92)	0.86 (0.80, 0.93)
D3*	0.87 (0.82, 0.92)	0.89 (0.84, 0.95)	0.89 (0.84, 0.95)	0.90 (0.85, 0.97)	0.89 (0.85, 0.94)	0.94 (0.89, 0.99)	0.94 (0.89, 1.00)	0.95 (0.89, 1.00)

*OR* odd ratio; *CI* confidence interval

^a^Univariate model

^b^Multivariable model adjusted for age and gender

^c^Adjusted for covariates in Model I plus marital status and educational level

^d^Adjusted for covariates in Model II plus smoking status, alcohol drinking status, BMI, number of chronic diseases and history of pneumonia or falls within the previous year

*D1: Ability to seek medical advice

D2: Seeking and appraising medical information

D3: Social resource support

**Fig. 1:**
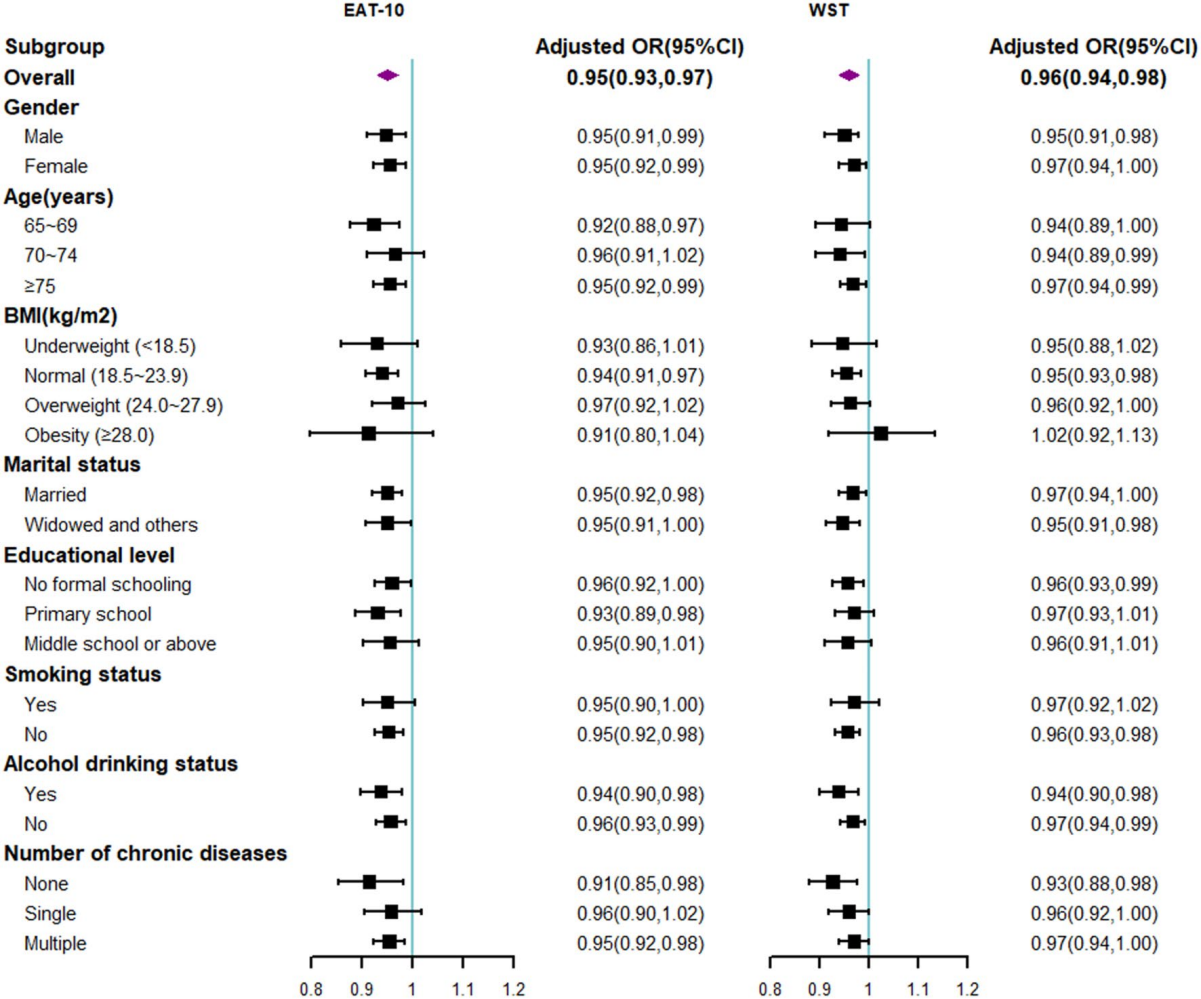
Subgroup analyses of association between health literacy and dysphagia. * The analysis was adjusted for various factors including age, gender, marital status, educational level, smoking status, alcohol drinking status, BMI, number of chronic diseases, and history of pneumonia or falls within the previous year

After excluding missing values in WST/EAT-10, CHLS, and the covariates (n = 4193 in the EAT-10 group, excluded 269; n = 4166 in the WST group, excluded 269), subgroup analyses were conducted according to gender, age, BMI, marital status, educational level, smoking status, alcohol drinking status, and the number of chronic diseases (Fig. 1) (detailed data shown in supplementary materials Table S2). Subgroup analysis by gender, marital status, and alcohol drinking status also revealed a significant association between low HL levels and dysphagia. In the age, BMI, educational level, smoking status, and the number of chronic diseases subgroups, the association between HL and dysphagia was significant only for participants aged 75 and above (adjusted OR = 0.95, 95% CIs 0.92–0.99 in EAT-10; adjusted OR = 0.97, 95% CIs 0.94–0.99 in WST), those with a normal BMI (adjusted OR = 0.94, 95% CIs 0.91–0.97 in EAT-10; adjusted OR = 0.95, 95% CIs 0.93–0.98 in WST), those who had no formal schooling (adjusted OR = 0.96, 95% CIs 0.92–1.00 in EAT-10; adjusted OR = 0.96, 95% CIs 0.93–0.99 in WST), non-smokers (adjusted OR = 0.95, 95% CIs 0.92–0.98 in EAT-10; adjusted OR = 0.96, 95% CIs 0.93–0.98 in WST), and those without chronic diseases (adjusted OR = 0.91, 95% CIs 0.85–0.98 in EAT-10; adjusted OR = 0.93, 95% CIs 0.88–0.98 in WST).

## Discussion

In this study, we found that dysphagia, as screened by the EAT-10 questionnaire and 30 ml-WST, was associated with HL. Moreover, we found a negative correlation between HL and dysphagia, indicating that older adults with dysphagia have lower levels of HL, affecting their ability to seek medical advice, acquire and assess medical information, and access social resources. These findings remained significant even after adjusting for various factors such as age, gender, marital status, educational level, BMI, history of pneumonia or falls within the previous year, and the number of chronic diseases. Subgroup analyses further revealed that this negative correlation was observed primarily among relatively healthy older adults who followed a healthy lifestyle.

Older adults with dysphagia may lack the ability to seek medical advice, possibly due to a lack of awareness regarding dysphagia symptoms and their acceptance of it as an inevitable aspect of aging [16]. Additionally, research suggests a significant number of individuals experiencing dysphagia do not seek help from healthcare professionals [17]. Consequently, older adults with lower HL are more prone to developing dysphagia and its associated serious consequences. Knowledge and skills play a crucial role in self-management, as individuals armed with greater information are empowered to participate in decision-making and make informed health choices [23]. Conversely, individuals with lower HL tend to rely less on advice from healthcare professionals but are more likely to trust friends, social media, or television, which may provide inadequate health information [24]. Thus, due to their limited ability to seek and assess medical information, older adults may possess insufficient knowledge about dysphagia, making it challenging to identify, intervene, and manage the condition in its early stages. Moreover, research suggests that social support significantly impacts the health outcomes of older adults [25]. Caregivers can encourage older adults to adopt healthy behaviors and lifestyles to manage their chronic diseases [26]. Consequently, older adults with robust social support are more inclined to engage in physical activities, maintain a healthy diet, and actively participate in social activities, thereby reducing the incidence and progression of chronic diseases and potentially reducing the occurrence of dysphagia.

Furthermore, subgroup analysis by gender, marital status, and alcohol drinking status indicated a significant association between low levels of HL and dysphagia. However, within the subgroup analysis based on BMI, this significance only persisted in the normal BMI group. This observation may be attributed to the fact that older adults who are underweight are already susceptible to dysphagia. Dysphagia leads to reduced food and liquid intake, and some older adults may even avoid eating due to fear of coughing, which can contribute to malnutrition and weight loss. Tran et al. [27] confirmed that malnutrition independently predicts the risk of dysphagia. On the other hand, this finding suggests that the protective effect of HL against dysphagia is more prominent in older adults with a normal BMI. Underweight individuals often experience poor nutritional status, which can lead to decreased muscle strength and weakness, thereby further compromising swallowing capacity. Conversely, overweight or obese individuals are more likely to have an unhealthy lifestyle, characterized by insufficient exercise and an unhealthy diet. This lifestyle may contribute to other diseases that impact the relationship between HL and dysphagia, explaining the change in significance within the overweight and obese subgroups.

Moreover, the protective effect of HL on dysphagia disappeared within the subgroup of individuals with a higher education level and was only observed among those who had no formal schooling. This discrepancy may arise from the association between education level and HL. Ganguli et al. [28] discovered that older adults with lower HL tend to have lower educational attainment. Individuals with higher education levels possess the ability to actively acquire, comprehend, and evaluate health information. Therefore, for older adults with lower education levels, improving their HL could help address their swallowing problems. Similarly, this effect only manifested in a relatively healthier population without chronic diseases. On one hand, older adults with chronic diseases experience poorer physical conditions, and conditions such as Parkinson's disease and stroke can lead to swallowing disorders. On the other hand, individuals with chronic diseases often require multiple medications, which can cause dysphagia by affecting esophageal peristalsis, reducing saliva secretion, impairing the coordination of swallowing muscles, and so on [29]. The presence of these factors might contribute to the disappearance of the HL effect on dysphagia. Additionally, the change in significance may be attributed to a smaller sample size following stratified analysis, resulting in lower effect values (detailed data can be found in supplementary materials Table S2).

This study has provided confirmation of a negative association between HL and dysphagia, which aligns with the notion that HL contributes to better management of chronic diseases [30]. Given the projected increase in the number of individuals living with dysphagia in their later years, improved outcomes can be achieved by leveraging HL. This underscores the need for policymakers and healthcare professionals to disseminate information on dysphagia, including strategies for prevention of complications, in order to mitigate the risk of aspiration, address potential issues, and enhance overall quality of life. Future prospective studies focusing on the relationship between HL and dysphagia can build upon the insights gained from this cross-sectional investigation.

Our study possessed several strengths. Firstly, it was a large-scale cross-sectional study involving 4,462 participants, allowing us to establish and affirm the association between HL and dysphagia. Additionally, we employed both subjective and objective tools to evaluate dysphagia, thereby minimizing potential biases. The newly developed HL measurement tool not only assessed comprehension and utilization of health information but also gauged the availability of social resources, enabling a more comprehensive identification of issues and the implementation of targeted interventions for improved dysphagia management. However, certain limitations must be acknowledged. Firstly, the study was conducted solely within a single community, potentially limiting the generalizability of the findings. Furthermore, the participants comprised relatively healthy older adults, introducing the possibility of selection bias. Moreover, as a prevalence study, it cannot establish a causal relationship between health literacy and dysphagia.

## Conclusions

In conclusion, our study demonstrated an inverse association between health literacy and dysphagia among older adults residing in the community. This association remained significant even after accounting for confounding factors. Effective interventions should be implemented to provide comprehensive support for community-dwelling older adults in terms of both medical services and social resources.

### Supplementary Information

Below is the link to the electronic supplementary material.

Supplementary file1 (DOCX 24 KB)

## Data Availability

The data that support the findings of this study are available from the corresponding authors, upon reasonable request.
