# Supplementary material for: Association between health literacy and dysphagia in the community-dwelling older population: a cross-sectional study
Source: Aging Clin Exp Res. 2023 Jul 28;35(10):2165–72. doi: 10.1007/s40520-023-02499-4 (PMC10520086; doi:10.1007/s40520-023-02499-4)
Supplement: Supplementary file 1 — Supplementary file1 (DOCX 24 KB) [file 40520_2023_2499_MOESM1_ESM.docx]

**Supplementary Materials**

**Association between Health Literacy and Dysphagia in the Community-Dwelling Older Population: A Cross-Sectional Study**

Table S1. The Chinese Health Literacy Scale (CHLS)

Table S2. Subgroup analysis of association between health literacy and dysphagia

Table S1 the Chinese Health Literacy Scale (CHLS)

| Dimensions | Items |
| --- | --- |
| D1: Ability to Seek Medical Advice | Item 4: When you are sick or in need of help, do you have many people you can rely on?  Item 6: Whether your problem is well understood by health care providers, you can effectively discuss health issues with them and obtain the health care information you need?  Item 7: Can you search and find the health care services that are best for you?  Item 9: Can you fill out medical forms, read and understand written information such as drug label information, and follow the doctor's instructions? |
| D2: Seeking and Appraising Medical Information | Item 5: Do you know how to verify health care information and compare the differences?  Item 8: Whether you have access to the latest, best and easy-to-understand health care information from multiple sources. |
| D3: Social Resource Support | Item 1: Is there at least one health care provider who knows you well enough to discuss health issues with you and guide you on what you need to do?  Item 2: Do you have enough information to maintain your health?  Item 3: Do you make plans, set goals, and take action to stay healthy? |

| Table S2 Subgroup analysis of association between health literacy and dysphagia | | | | | | |
| --- | --- | --- | --- | --- | --- | --- |
|  | EAT-10 | | | WST | | |
|  | n | Adjusted OR(95%CI) | p | n | Adjusted OR(95%CI) | p |
| Overall | 4193 | 0.95(0.93,0.97) | **<0.05** | 4166 | 0.96(0.94,0.98) | **<0.05** |
| Subgroup |  |  |  |  |  |  |
| Gender |  |  |  |  |  |  |
| Male | 2078 | 0.95(0.91,0.99) | **<0.05** | 2063 | 0.95(0.91,0.98) | **<0.05** |
| Female | 2115 | 0.95(0.92,0.99) | **<0.05** | 2103 | 0.97(0.94,1.00) | **<0.05** |
| Age(years) |  |  |  |  |  |  |
| 65~69 | 1413 | 0.92(0.88,0.97) | **<0.05** | 1404 | 0.94(0.89,1.00) | **0.05** |
| 70~74 | 1338 | 0.96(0.91,1.02) | 0.21 | 1325 | 0.94(0.89,0.99) | **<0.05** |
| ≥75 | 1442 | 0.95(0.92,0.99) | **<0.05** | 1437 | 0.97(0.94,0.99) | **<0.05** |
| BMI(kg/m^2^) |  |  |  |  |  |  |
| Underweight (<18.5) | 225 | 0.93(0.86,1.01) | 0.07 | 227 | 0.95(0.88,1.02) | 0.12 |
| Normal (18.5~23.9) | 2265 | 0.94(0.91,0.97) | **<0.05** | 2257 | 0.95(0.93,0.98) | **<0.05** |
| Overweight (24.0~27.9) | 1370 | 0.97(0.92,1.02) | 0.26 | 1353 | 0.96(0.92,1.00) | 0.07 |
| Obesity (≥28.0) | 333 | 0.91(0.80,1.04) | 0.18 | 329 | 1.02(0.92,1.13) | 0.66 |
| Marital status |  |  |  |  |  |  |
| Married | 3252 | 0.95(0.92,0.98) | **<0.05** | 3231 | 0.97(0.94,1.00) | **<0.05** |
| Widowed and others | 941 | 0.95(0.91,1.00) | **<0.05** | 935 | 0.95(0.91,0.98) | **<0.05** |
| Educational level |  |  |  |  |  |  |
| No formal schooling | 1196 | 0.96(0.92,1.00) | **<0.05** | 1199 | 0.96(0.93,0.99) | **<0.05** |
| Primary school | 1784 | 0.93(0.89,0.98) | **<0.05** | 1765 | 0.97(0.93,1.01) | 0.14 |
| Middle school or above | 1213 | 0.95(0.90,1.01) | 0.10 | 1202 | 0.96(0.91,1.01) | 0.07 |
| Smoking status |  |  |  |  |  |  |
| Yes | 1258 | 0.95(0.90,1.00) | 0.06 | 1243 | 0.97(0.92,1.02) | 0.22 |
| No | 2935 | 0.95(0.92,0.98) | **<0.05** | 2923 | 0.96(0.93,0.98) | **<0.05** |
| Alcohol drinking status |  |  |  |  |  |  |
| Yes | 1516 | 0.94(0.90,0.98) | **<0.05** | 1505 | 0.94(0.90,0.98) | **<0.05** |
| No | 2677 | 0.96(0.93,0.99) | **<0.05** | 2661 | 0.97(0.94,0.99) | **<0.05** |
| Number of chronic diseases |  |  |  |  |  |  |
| None | 698 | 0.91(0.85,0.98) | **<0.05** | 688 | 0.93(0.88,0.98) | **<0.05** |
| Single | 1349 | 0.96(0.90,1.02) | 0.14 | 1343 | 0.96(0.92,1.00) | **0.05** |
| Multiple | 2146 | 0.95(0.92,0.98) | **<0.05** | 2135 | 0.97(0.94,1.00) | **0.05** |
